# Strategies for Multiplexing Plasmonic Biosensing

**DOI:** 10.3390/s26154964

**Published:** 2026-08-05

**Authors:** Muhammad Umair Khan, Jaroslav Katrlík

**Affiliations:** Institute of Chemistry, Slovak Academy of Sciences, Dúbravska cesta 5807/9, 845 38 Bratislava, Slovakia; umair.khan@savba.sk

**Keywords:** plasmonic biosensing, multiplexed biosensing, surface plasmon resonance, surface plasmon resonance imaging (SPRi), localized surface plasmon resonance, surface-enhanced Raman scattering, microarrays, microfluidics, glycoprofiling, extracellular vesicles, liquid biopsy, food safety, environmental monitoring, artificial intelligence

## Abstract

Plasmonic biosensing technologies have emerged as powerful analytical tools for sensitive and label-free characterisation of biomolecular interactions and complex samples. The increasing demand for comprehensive molecular profiling has accelerated the development of multiplexing strategies that enable simultaneous analysis of multiple analytes and molecular interactions. This Feature Paper examines multiplexing through the complementary spatial, spectral, and temporal dimensions of multiplexing, together with their hybrid combinations and associated analytical trade-offs. Compared with other optical biosensing approaches, including interferometric, photonic, and fluorescence-based sensing platforms, plasmonic biosensors remain attractive owing to their combination of label-free detection, real-time interaction monitoring, sensitive interfacial analysis, and compatibility with multiplexed assay formats. This Feature Paper critically discusses current multiplexing strategies, focusing primarily on surface plasmon resonance (SPR), imaging SPR (SPRi), localised SPR (LSPR), surface-enhanced Raman scattering (SERS), and related nanoplasmonic biosensing approaches, together with recent advances in surface biofunctionalisation, antifouling interfaces, and molecular recognition strategies. Representative applications in biomedical diagnostics and non-clinical settings are highlighted, with examples such as liquid biopsy, glycoprofiling, extracellular vesicle profiling, and food and environmental analysis, alongside key challenges in reproducibility, standardisation, data interpretation, and clinical translation. In addition, selected non-plasmonic optical biosensing technologies are briefly discussed to position plasmonic biosensing within the broader landscape of multiplexed optical biosensing. This Feature Paper argues that the future of multiplexed plasmonic biosensing will depend less on further improvements in sensor performance than on robust, standardised analytical systems.

## 1. Introduction: The Unmet Need for Multiplexed Plasmonic Biosensing

The ongoing transition from empirical and reductionist approaches towards systems-level and data-driven bioanalysis has substantially increased the demand for analytical technologies capable of capturing biological complexity at multiple molecular levels.

It is now widely recognised that many biological processes and diseases, including cancer, inflammatory disorders, neurodegenerative diseases, and infectious diseases, cannot be adequately described by a single biomarker. Similar analytical challenges also arise in food safety and environmental monitoring, where reliable decisions increasingly depend on the simultaneous detection and interpretation of multiple molecular targets. This paradigm shift has stimulated the development of multiplexed biosensing technologies capable of simultaneously analysing multiple molecular targets while reducing sample consumption, assay time, and experimental variability. Such approaches are especially relevant in liquid biopsy applications, where biological heterogeneity often necessitates parallel assessment of multiple biomarkers rather than reliance on single-analyte measurements. Beyond biomedical diagnostics, multiplexed analytical strategies are increasingly being applied to the simultaneous detection of foodborne pathogens, toxins, chemical contaminants, and environmental pollutants, thereby enabling more comprehensive analysis of complex biological and non-biological samples [1,2,3,4].

Within the broader landscape of optical biosensing technologies, including fluorescence-based, interferometric, and photonic sensing approaches, plasmonic biosensors occupy a distinctive position. Surface plasmon resonance (SPR) and its derivatives, including imaging SPR (SPRi), localised surface plasmon resonance (LSPR), and surface-enhanced Raman scattering (SERS), have evolved from biomolecular interaction analysis tools into versatile analytical platforms suitable for multiplexed biosensing. Concurrent advances in nanotechnology, microfluidics, surface engineering, and biointerface design have further expanded their capabilities towards high-content molecular profiling and translational applications [5].

Despite substantial technological progress, routine implementation of multiplexed plasmonic biosensing remains limited. Current challenges extend beyond analytical sensitivity and include assay reproducibility, standardisation of biofunctionalisation protocols, non-specific interactions, inter-platform comparability, data interpretation, and integration into established workflows. Increasing assay dimensionality has also generated a growing need for data-driven analytical approaches and machine learning-assisted interpretation of complex biosensing datasets. Importantly, many promising plasmonic platforms remain at the proof-of-concept stage, highlighting a persistent gap between laboratory demonstrations and robust real-world implementation [6,7,8].

Against this background, this Feature Paper discusses strategies for multiplexing plasmonic biosensing from a conceptual and translational perspective. Particular emphasis is placed on multiplexing paradigms implemented in SPR, SPRi, LSPR, SERS, and related nanoplasmonic platforms, together with enabling surface biofunctionalisation strategies, antifouling interfaces, and molecular recognition approaches. Emerging applications in biomedical diagnostics, food, and environmental analysis are critically discussed alongside current barriers to standardisation and translation. Selected non-plasmonic optical biosensing technologies are also considered to position plasmonic biosensing within the broader context of multiplexed optical biosensing and to identify realistic future opportunities for the field.

### 1.1. From Single-Analyte Measurements to Multiplexed Molecular Profiling

The increasing adoption of multiplexed analytical technologies is fundamentally changing how complex analytical information is generated and interpreted. Rather than relying on individual biomarkers or analytes, contemporary biomedical research, clinical diagnostics, and non-clinical analytical applications increasingly seek to characterise coordinated molecular signatures or multiple analytical targets simultaneously. As a result, the analytical value of multiplexing lies not simply in measuring more targets, but in integrating complementary information that reveals relationships and patterns inaccessible to single-analyte approaches.

Oncology provides one of the clearest examples of this paradigm shift. Tumours exhibit substantial inter- and intra-tumour heterogeneity at genomic, transcriptomic, proteomic, and metabolic levels, which severely limits the utility of individual biomarkers for early diagnosis and patient stratification. For example, carbohydrate antigen 19-9 (CA19-9), the only clinically established blood biomarker for pancreatic ductal adenocarcinoma, exhibits a pooled sensitivity of approximately 79% and specificity of 82%, highlighting the limitations of relying on a single marker for disease surveillance. Consequently, increasing efforts are directed towards multi-analyte biomarker panels and multi-omics approaches that integrate complementary molecular information [2].

Similar trends are emerging in inflammatory and infectious diseases. Cytokines, chemokines, and other inflammatory mediators operate within highly interconnected regulatory networks, while syndromic infectious disease panels increasingly rely on simultaneous detection of multiple pathogens and host-response markers. Such approaches provide a more comprehensive description of disease states than individual biomarkers alone and are becoming important components of modern biomedical research and clinical practice [3,9].

Comparable analytical requirements are also emerging outside biomedical research. In food analysis, simultaneous detection of multiple pathogens, toxins, allergens, or chemical contaminants provides more comprehensive information than individual assays. Similarly, environmental monitoring increasingly relies on multiplexed detection of pollutants, microorganisms, and other indicators to capture the complexity of environmental systems and improve analytical reliability.

### 1.2. Why Plasmonics Are Well Suited for Multiplexed Biosensing

Plasmonic biosensors occupy a distinctive position within optical biosensing because they combine several properties that are especially valuable for multiplexed bioanalysis: surface-sensitive optical transduction, label-free and real-time interaction monitoring, and compatibility with patterned, microarray-based, and microfluidic assay formats. None of these features is entirely exclusive to plasmonics; interferometric, photonic, and fluorescence-based platforms can also support sensitive and multiplexed measurements. The practical strength of plasmonic biosensing lies instead in the well-established combination of these capabilities within experimentally mature and broadly adaptable sensing formats [10,11].

Surface plasmon resonance (SPR) remains the most established plasmonic modality for label-free biomolecular interaction analysis. By monitoring refractive-index changes near a functionalised metal–dielectric interface, SPR enables real-time measurement of association and dissociation processes without the need for fluorescent or enzymatic labels. This makes SPR well suited to interaction-centric applications that benefit from simultaneous access to concentration, affinity, and kinetic information. Imaging SPR (SPRi) extends these capabilities to spatially resolved microarray formats, allowing multiple ligands or capture probes to be monitored in parallel on a single sensor surface [12,13,14].

Localised surface plasmon resonance (LSPR) platforms provide a complementary route towards miniaturised and highly adaptable multiplexed plasmonic sensing. Because the optical response is generated by nanostructured metallic features rather than a continuous metal film, LSPR sensors are well suited to miniaturised, patterned, and potentially low-volume assay formats. Their shorter sensing depth can be advantageous for detecting surface-proximal binding events, although this simultaneously increases the importance of robust surface functionalisation and control of non-specific interactions. Multiplexed LSPR imaging and nanoplasmonic array formats therefore offer attractive opportunities, but their analytical robustness is strongly influenced by nanostructure quality, surface chemistry, and nanofabrication reproducibility [15,16].

Surface-enhanced Raman scattering (SERS) introduces an orthogonal multiplexing dimension by exploiting molecular spectroscopic fingerprints instead of refractive-index changes. This makes SERS particularly powerful for high-content and multi-marker assays, but also introduces challenges related to substrate reproducibility, quantitative calibration, reporter design, and signal variability. Therefore, SERS should be viewed as a complementary plasmonic modality, not only as a direct equivalent of SPR-based kinetic biosensing.

Rather than representing a single sensing technology, plasmonic biosensing should be regarded as a family of complementary analytical frameworks encompassing modalities with distinct multiplexing capabilities. Throughout this Feature Paper, multiplexing is therefore discussed not as an intrinsic property of any single plasmonic modality, but as a set of analytical strategies that can be implemented across SPR-, SPRi-, LSPR-, and SERS-based systems. Understanding the strengths, limitations, and complementarities of these modalities is essential for identifying realistic pathways towards robust, reproducible, and translational implementations of multiplexed plasmonic biosensing.

### 1.3. Why Multiplexing Remains Challenging

The same characteristics that make plasmonic biosensors attractive for multiplexed bioanalysis also impose fundamental constraints on assay design, analytical robustness, and data interpretation. Importantly, multiplexing should not be regarded as a straightforward scaling-up of single-analyte biosensing. Increasing multiplexing levels fundamentally alters how sensing surfaces are engineered, how signals are extracted and interpreted, and how analytical performance is maintained across increasingly complex assay architectures. Consequently, successful multiplexed plasmonic biosensing requires coordinated optimisation of sensor design, surface chemistry, assay configuration, and computational analysis rather than improvements in any individual component alone [17].

Four interrelated challenge domains are central to successful multiplexing: signal deconvolution, surface engineering, assay specificity, and the inherent trade-offs associated with increasing multiplexing density. Although their relative importance varies across plasmonic modalities, these challenges are common to virtually all multiplexed implementations and ultimately define the practical design space of multiplexed plasmonic biosensors.

Signal deconvolution becomes increasingly demanding as multiplexing density increases because multiple optical, spectral, or spatially resolved signals must be separated without introducing cross-talk between neighbouring sensing channels. In SPR and SPRi platforms, signal interpretation can be complicated by non-specific adsorption, mass transport effects, and spatial interference between adjacent sensing regions. In LSPR systems, broad nanoparticle extinction spectra may limit spectral encoding capacity, whereas SERS-based assays can suffer from overlapping Raman signatures and heterogeneous electromagnetic enhancement. Consequently, multiplexing performance is often constrained not by signal generation itself, but by the ability to robustly separate and interpret increasingly complex datasets [15,17].

Surface engineering constitutes a second fundamental challenge. Multiplexed biosensors require multiple recognition elements to coexist on a limited sensing area while maintaining high accessibility, specificity, and reproducibility. Increasing probe density may lead to steric hindrance, reduced target accessibility, and greater susceptibility to non-specific adsorption and biofouling, particularly in complex biological matrices. These effects become increasingly important when analysing large biological entities, including extracellular vesicles (EVs) and other supramolecular assemblies, whose dimensions approach the characteristic length scales of the sensing interface. Therefore, surface chemistry is not merely a supporting component of multiplexed biosensing, but a central determinant of assay performance [9,18].

Maintaining assay specificity also becomes progressively more difficult as multiplexing levels increase. Cross-reactivity between recognition elements, unintended interactions with matrix components, and non-specific adsorption can all compromise analytical accuracy. These effects are particularly pronounced in clinical samples, where the large dynamic range and molecular complexity of biological fluids challenge both assay design and data interpretation. Consequently, achieving robust multiplexed measurements requires careful optimisation of both recognition chemistries and antifouling interfaces [17,18].

Finally, multiplexed biosensing is governed by a series of unavoidable analytical trade-offs. Increasing the number of simultaneously measured targets generally improves information content but may reduce sensitivity, increase signal variability, and complicate data interpretation. Similarly, high-density arrays can improve throughput while reducing the active sensing area available to individual capture probes. Rather than representing technological shortcomings, these trade-offs reflect the intrinsic complexity of multiplexed bioanalysis and necessitate careful balancing between throughput, sensitivity, reproducibility, and assay robustness.

These considerations highlight that multiplexing is not an intrinsic property of plasmonic biosensors, but a systems-level analytical challenge. Future progress will depend on integrated solutions that combine advanced surface engineering, microfluidic architectures, hybrid multiplexing strategies, and computational methods, including machine learning-assisted data analysis. This perspective forms the conceptual basis for the subsequent sections of this Feature Paper, which critically discuss how different plasmonic modalities implement and address these challenges.

### 1.4. Scope and Objectives of This Feature Paper

This Feature Paper is not intended to provide an exhaustive technology-centred review of plasmonic biosensing. Instead, it aims to deliver a critical and conceptually driven synthesis of the principal strategies currently employed to achieve multiplexing in plasmonic biosensing, with particular emphasis on their analytical strengths, practical limitations, and translational potential. The focus is therefore on identifying common design principles, unresolved challenges, and realistic opportunities for future progress rather than on cataloguing individual technological implementations.

The literature discussed in this Feature Paper was selected to provide a representative and critical overview of major developments relevant to multiplexing strategies in plasmonic biosensing, with particular emphasis on recent advances, influential studies, and illustrative examples that support the conceptual framework presented throughout the manuscript. Accordingly, this article should not be regarded as a systematic review of the literature. Rather than providing exhaustive technical coverage of individual plasmonic platforms, this Feature Paper deliberately adopts this broad, concept-driven perspective that facilitates comparison across multiple sensing modalities and application areas. This approach is intended to establish a unifying analytical framework that identifies common analytical principles, shared design trade-offs, and translational challenges extending across plasmonic technologies and application areas.

Three overarching perspectives guide this analysis. First, multiplexing is approached primarily as an analytical concept rather than as a collection of independent technological solutions. Accordingly, spatial, spectral, temporal, and hybrid multiplexing strategies are discussed as complementary paradigms that can be implemented across different plasmonic modalities. Second, particular emphasis is placed on biointerfaces, including surface biofunctionalisation, antifouling strategies, and molecular recognition chemistries, as these often determine the practical success of multiplexed biosensors when analysing biological samples. Third, multiplexing strategies are evaluated in the context of biomedical and non-clinical translational applications.

An additional objective is to position plasmonic biosensing within the wider field of multiplexed optical bioanalysis. To this end, selected non-plasmonic optical technologies, including fluorescence-based, interferometric, and photonic approaches, are briefly discussed to provide analytical context rather than a comprehensive comparison. Computational approaches, including machine learning and AI-assisted analysis, are also considered as emerging enabling tools for addressing the increasing complexity of multiplexed datasets.

The structure of this Feature Paper follows a concept-to-translation framework. Section 2 introduces the fundamental paradigms of multiplexing and establishes a conceptual basis for comparing different plasmonic implementations. Section 3 critically discusses current multiplexing strategies across SPR, SPRi, LSPR, SERS, and emerging nanoplasmonic platforms. Section 4 focuses on the enabling role of biointerfaces and molecular recognition strategies. Section 5 highlights translational applications in liquid biopsy, glycoprofiling, EVs profiling, precision diagnostics, food, and environmental analyses. Section 6 positions plasmonic biosensing within the broader optical multiplexing landscape, while Section 7 addresses challenges related to reproducibility, standardisation, data interpretation, and translation. Finally, Section 8 discusses future perspectives, including data-driven and AI-assisted biosensing approaches. Collectively, this structure aims to provide a realistic and application-oriented framework for understanding the current state and future direction of multiplexed plasmonic biosensing. Such a conceptual framework is essential because meaningful comparison of plasmonic multiplexing strategies requires common analytical criteria rather than technology-specific evaluation.

## 2. A Conceptual Framework for Multiplexed Plasmonic Biosensing

This section establishes a conceptual framework for comparing multiplexing strategies across plasmonic biosensing. Rather than cataloguing individual technological implementations, it defines a common analytical language that forms the basis for the discussions that follow.

### 2.1. What Constitutes Multiplexing?

In this Feature Paper, multiplexing is defined as the simultaneous or orthogonally resolved analysis of two or more molecular targets within a single measurement cycle using a common sensing interface. This definition intentionally excludes independent single-analyte measurements performed in separate experiments or on different instruments, as the principal advantages of multiplexing arise from integrating multiple measurements within a unified analytical workflow.

**Figure 1:**
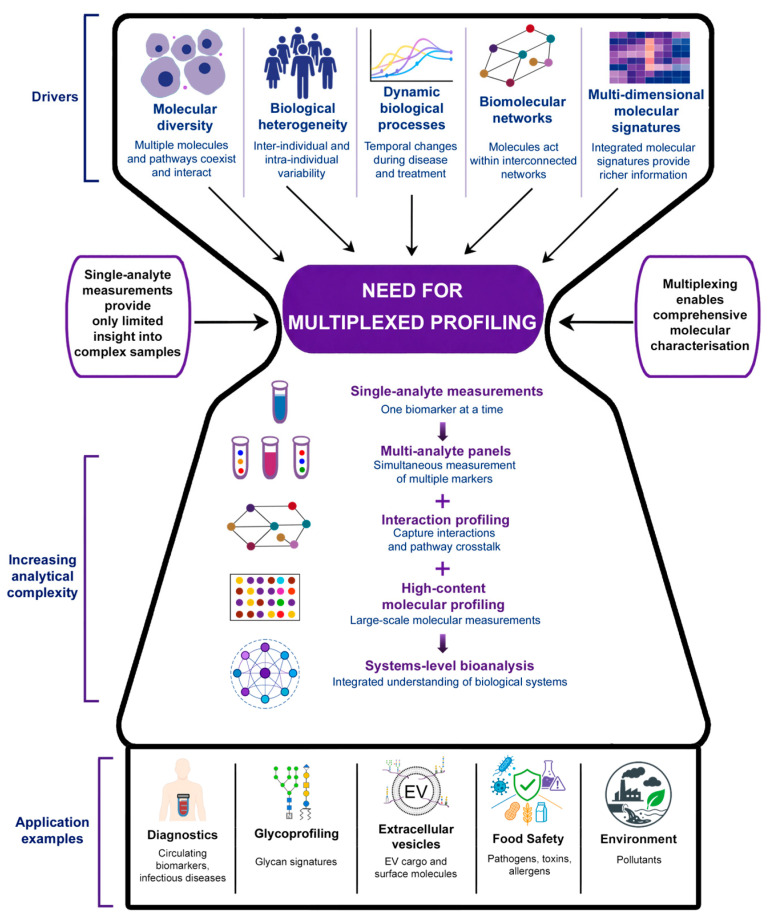
Conceptual evolution from single-analyte measurements to multiplexed profiling. Increasing biological complexity, including molecular diversity, biological heterogeneity, dynamic biological processes, biomolecular networks, and multi-dimensional molecular signatures, drives the need for multiplexed molecular profiling. Analytical approaches evolve from single-analyte measurements towards interaction profiling and high-content molecular analysis, ultimately enabling systems-level bioanalysis. Representative translational applications include diagnostics, glycoprofiling, EVs profiling, food analysis, and environmental monitoring.

Importantly, multiplexing should not be confused with miniaturisation, high-throughput screening, or simple parallelisation. Although these concepts frequently coexist, multiplexing specifically refers to the ability to extract multidimensional molecular information from a single experiment while preserving analytical context between measured targets. Table 1 summarises the principal analytical drivers that have accelerated the transition from single-analyte measurements towards multiplexed molecular profiling, and Figure 1 illustrates the conceptual evolution from single-analyte measurements towards multidimensional molecular profiling.

**Figure 2:**
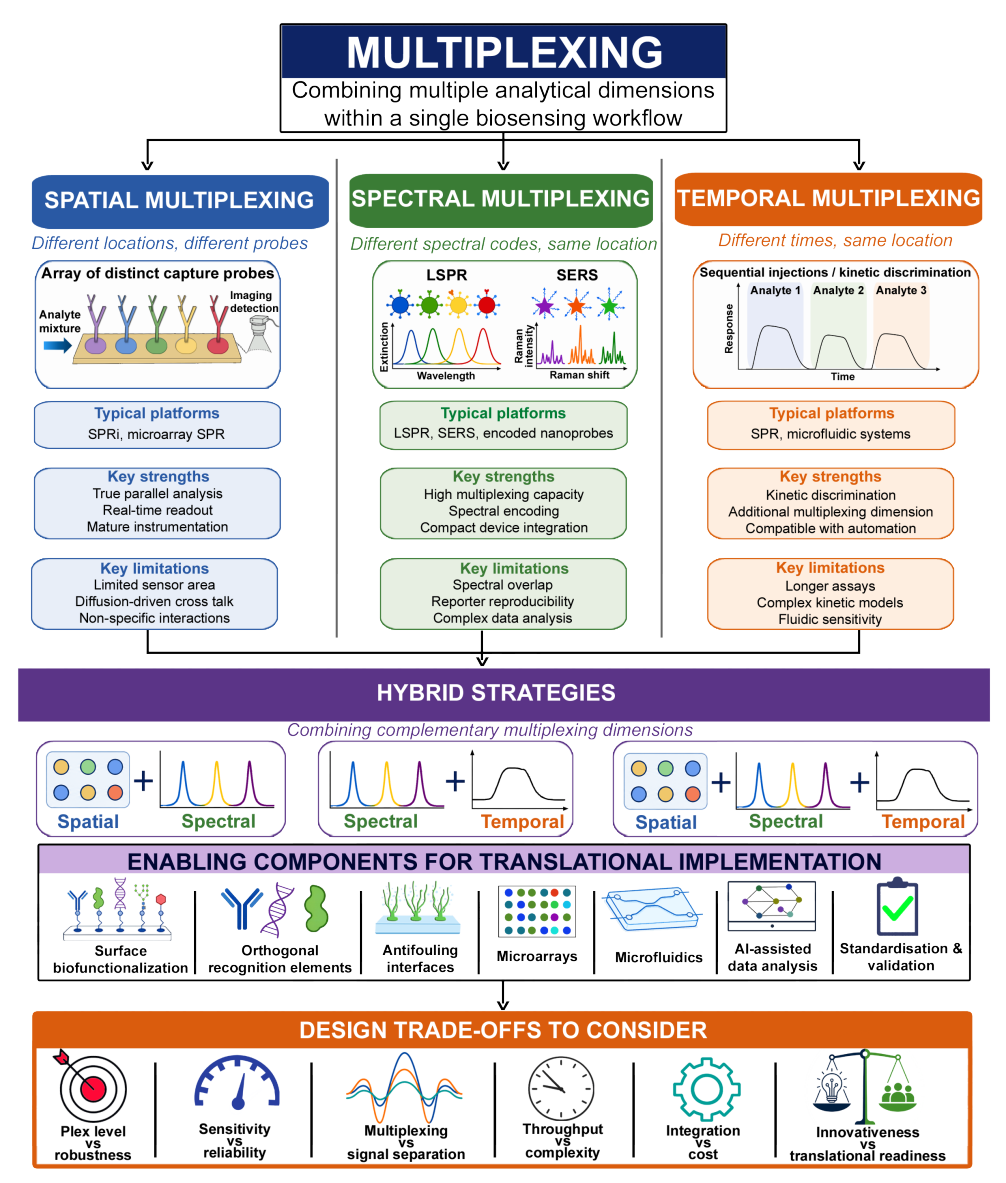
Conceptual framework of multiplexing strategies in plasmonic biosensing.

Multiplexing can be implemented along three principal analytical dimensions: spatial, spectral, and temporal. These dimensions may be combined into hybrid strategies that increase information content while simultaneously introducing trade-offs related to sensitivity, throughput, assay complexity, reproducibility, and translational readiness. Successful implementation also depends on enabling components, including molecular recognition elements, biointerface engineering, microfluidic integration, and microarray architectures. The figure highlights multiplexing as a multidimensional design space rather than a single technological attribute. Figure 2 summarises this conceptual framework.

### 2.2. Fundamental Multiplexing Paradigms

Most multiplexed plasmonic biosensors can be described using three fundamental paradigms: spatial, spectral, and temporal multiplexing.

Spatial multiplexing relies on physically separated sensing regions, each functionalised with a distinct recognition element. During the measurement, signals are assigned to predefined locations on the sensor surface and simultaneously monitored using imaging-based detection systems. SPRi represents the most mature implementation of this concept and enables parallel monitoring of multiple interactions within a single experiment. The principal advantages of spatial multiplexing are its intuitive implementation and straightforward label-free readout. However, achievable multiplexing density remains constrained by available sensor area, analyte diffusion, and the need to minimise cross-talk between neighbouring sensing regions [15,18].

Spectral multiplexing encodes molecular identity in the wavelength or frequency domain. In LSPR, nanostructures with distinct plasmonic properties can generate spectrally distinguishable responses, whereas SERS exploits narrow Raman signatures or Raman reporter molecules to achieve high multiplexing levels. Spectral approaches offer substantial multiplexing capacity but become increasingly susceptible to spectral overlap, signal variability, and deconvolution challenges as multiplexing density increases [17,19].

Temporal multiplexing exploits differences in binding kinetics or controlled temporal separation of measurements. Sequential sample injection, microfluidic switching, and kinetic discrimination can provide an additional multiplexing dimension without increasing sensor density. Temporal multiplexing is currently less widespread than spatial and spectral implementations and is most often employed as an orthogonal multiplexing dimension that complements other strategies rather than replacing them [18].

Table 2 summarises representative implementations of multiplexing across major plasmonic platforms. Because multiplexing capacity cannot be represented by a single universal numerical metric, representative implementations span a broad range of practical multiplexing scales.

### 2.3. Hybrid Multiplexing Strategies and Design Implications

Future multiplexed plasmonic biosensors will increasingly rely on hybrid strategies that combine two or more multiplexing paradigms. Spatial–spectral approaches, for example, can substantially increase multiplexing density by assigning spectrally distinguishable signatures to individual sensing regions. Similarly, spatial–temporal implementations enable kinetic discrimination across multiple sensing locations, improving assay specificity and reducing the impact of non-specific interactions.

Hybridisation should be regarded as a design philosophy rather than a separate multiplexing category. The choice of multiplexing strategy directly influences sensor architecture, surface chemistry, microfluidic integration, and computational requirements. Spatial implementations require robust patterning and imaging capabilities; spectral implementations depend on reproducible reporter chemistry and signal deconvolution; temporal implementations require precise fluidic control and kinetic modelling.

Ultimately, no universal multiplexing strategy exists. Instead, optimal assay design depends on balancing multiplexing density, analytical robustness, reproducibility, and application-specific requirements. Recognising these trade-offs is essential for developing multiplexed plasmonic biosensors that are not only technologically sophisticated, but also analytically robust and translationally relevant. The following sections examine how these conceptual principles are implemented across the principal plasmonic sensing modalities, each of which represents a distinct solution to the common analytical challenges outlined above. This conceptual framework is intended to provide a common analytical basis for comparing multiplexing strategies rather than a prescriptive classification of plasmonic technologies.

## 3. Implementing Multiplexing Across Plasmonic Modalities

The conceptual framework introduced in Figure 2 facilitates systematic comparison of how individual plasmonic modalities implement multiplexing. Because individual plasmonic platforms frequently incorporate multiple multiplexing paradigms, the following discussion is organised according to the principal sensing modalities to facilitate comparison of their analytical capabilities within this conceptual framework. In practice, however, multiplexing is realised differently across plasmonic modalities, each offering distinct analytical strengths, limitations, and levels of technological maturity. Rather than representing competing technologies, SPR, SPRi, LSPR, SERS, and emerging nanoplasmonic platforms should be regarded as complementary approaches that occupy different positions within the multiplexing landscape. Their value lies not in maximising multiplexing density alone, but in balancing information content, analytical robustness, and translational applicability.

### 3.1. Conventional Surface Plasmon Resonance (SPR)

Within the conceptual framework introduced in Section 2, conventional SPR primarily represents the temporal and kinetic dimension of multiplexed plasmonic biosensing. Rather than maximising multiplexing density, its principal strength lies in providing robust real-time interaction analysis that serves as a reference for many subsequent multiplexed implementations. SPR should therefore not be regarded as a highly multiplexed platform *per se*, but rather as the reference technology from which many multiplexed plasmonic implementations have evolved. Owing to its mature instrumentation, robust surface chemistry, and unique ability to provide real-time kinetic information, SPR remains the benchmark platform for interaction-centric bioanalysis [10,13,20].

The inherent multiplexing capacity of conventional prism-based SPR is relatively limited. Most commercial instruments interrogate one or a few sensing channels simultaneously, and increasing multiplexing density rapidly introduces constraints associated with fluidic complexity and sensor architecture. Consequently, multiplexing in SPR is typically implemented through sequential sample injection, multi-channel flow cells, or integration with microfluidic systems rather than through high-density sensor arrays [17].

Despite these limitations, SPR remains especially valuable whenever multiplexing must be combined with quantitative kinetic analysis. Simultaneous access to concentration, affinity, and binding kinetics provides information that is difficult to obtain using endpoint assays and remains one of the principal advantages of SPR-based platforms. This capability is especially relevant for interaction profiling, assay validation, and discrimination between specific and non-specific binding events. Future developments are expected to focus on improved integration with microfluidics, enhanced automation, and coupling with complementary analytical modalities. In this context, SPR should be viewed as the kinetic reference platform within multiplexed plasmonic biosensing rather than as a direct competitor to high-density multiplexing technologies.

### 3.2. Imaging Surface Plasmon Resonance (SPRi)

Among the multiplexing paradigms introduced in Section 2, SPRi provides the most mature implementation of spatial multiplexing in plasmonic biosensing. By combining SPR with imaging-based detection, multiple sensing regions can be monitored simultaneously across a single sensor surface, effectively transforming the sensor into a label-free microarray platform [12,21]. Recent developments have further expanded the capabilities of SPRi towards highly parallel kinetic microarrays and multiplexed quantitative analyses of affinity, concentration, and specificity [22].

Different capture probes are immobilised at predefined locations on the gold surface, while CCD or CMOS cameras continuously monitor reflectivity changes associated with biomolecular interactions. This architecture enables simultaneous analysis of dozens to hundreds of interactions within a single experiment while preserving the real-time and label-free nature of conventional SPR.

The principal strength of SPRi lies in its ability to bridge interaction analysis and microarray technology. Commercial and research platforms have demonstrated simultaneous kinetic analysis across hundreds of sensing regions, illustrating how microarray concepts can be integrated with real-time and label-free biosensing [23]. Unlike conventional microarrays, which typically provide endpoint measurements, SPRi delivers dynamic information and therefore substantially richer analytical content.

Numerous studies have demonstrated multiplexed detection of diverse molecular targets, highlighting the considerable analytical and translational potential of SPRi-based platforms. Representative applications include high-content molecular profiling and integrated multi-analyte analysis, where simultaneous access to complementary molecular information may provide a more comprehensive representation of complex biological or analytical systems than single-analyte measurements alone [24].

However, increasing multiplexing density also introduces several challenges. Signal quality may be compromised by optical non-uniformities, spatial cross-talk, and reduced sensitivity compared with single-channel SPR. In addition, data analysis becomes progressively more demanding as array density increases. Consequently, future progress will depend on improved image processing, automated surface quality control, and integration with data-driven analytical approaches. SPRi remains one of the most versatile platforms and represents the benchmark for implementing spatial multiplexing.

### 3.3. Localised Surface Plasmon Resonance (LSPR)

Whereas SPRi expands multiplexing primarily through spatial parallelisation, LSPR extends it through miniaturisation and flexible spectral engineering, since it offers a fundamentally different route towards multiplexing by exploiting nanostructured metallic architectures instead of continuous metal films. Because optical responses originate from individual nanostructures, multiplexing is inherently embedded within many LSPR implementations rather than being added as an external functionality [15,16].

Two complementary strategies dominate current LSPR approaches. The first relies on spatially addressable nanoparticle arrays, where multiple sensing regions are patterned onto a single substrate. The second exploits spectral encoding, taking advantage of the fact that plasmonic responses depend strongly on nanoparticle size, shape, and composition. These strategies can also be combined within hybrid architectures.

Compared with SPR-based systems, LSPR offers several practical advantages. Its shorter sensing depth improves sensitivity towards surface-proximal binding events, while the absence of prism-based optics facilitates miniaturisation and integration into portable devices. Such properties make LSPR especially attractive for decentralised testing environments, portable analytical systems, and future point-of-care implementations. Recent advances have further demonstrated the potential of engineered nanoplasmonic architectures for highly integrated and multiplexed biosensing applications [25].

These advantages are accompanied by important challenges. Analytical performance remains highly dependent on nanofabrication reproducibility, nanoparticle heterogeneity, and surface functionalisation quality. Variability between nominally identical nanostructures can significantly influence spectral responses and complicate quantitative measurements. Consequently, standardisation of nanomaterial production remains one of the major obstacles to the broader implementation of LSPR biosensors. LSPR therefore occupies the interface between laboratory plasmonics and miniaturised translational biosensing.

### 3.4. Surface-Enhanced Raman Scattering (SERS)

SERS represents the clearest example of spectral multiplexing within the conceptual framework presented in Section 2. This technique occupies a unique position within multiplexed plasmonic biosensing because it introduces an orthogonal multiplexing dimension based on spectroscopic rather than refractive-index readouts. Instead of monitoring changes at the sensor interface, SERS exploits narrow Raman signatures generated by reporter molecules located within highly enhanced electromagnetic fields, commonly referred to as hotspots [26,27].

This approach offers exceptional multiplexing capacity. Multiple Raman reporters can be excited using a single laser wavelength while remaining spectrally distinguishable owing to their characteristic vibrational fingerprints. Multiplexing levels exceeding twenty simultaneously resolvable reporters have already been demonstrated under controlled experimental conditions, considerably surpassing the multiplexing capacity of most other plasmonic modalities [26,28]. The high information content of SERS has stimulated applications in molecular fingerprinting, immune profiling, pathogen identification, and multiplexed biomarker analysis. Especially in oncology and infectious disease diagnostics, SERS offers an attractive combination of sensitivity and multiplexing capacity that is difficult to achieve using other plasmonic approaches [29,30].

However, this exceptional multiplexing capability is accompanied by important practical limitations. Quantitative analysis remains difficult because signal intensities depend strongly on hotspot distribution, substrate uniformity, and reporter chemistry. Batch-to-batch variability and limited standardisation further complicate reproducibility and clinical translation. Consequently, current research increasingly focuses on improving substrate manufacturing, calibration strategies, and inter-laboratory reproducibility, recognising that these advances are essential for broader analytical and clinical adoption. SERS should not be regarded as a direct alternative to SPR-based kinetic biosensing, but as a complementary modality that excels in high-content molecular profiling. SERS therefore defines the upper limit of spectral information density currently achievable in plasmonic biosensing.

### 3.5. Emerging Nanoplasmonic and Hybrid Approaches

Rather than introducing an entirely new multiplexing paradigm, emerging plasmonic platforms increasingly combine spatial, spectral, temporal, and computational strategies into integrated analytical systems, thus blurring the boundaries between conventional plasmonics, nanophotonics, and computational bioanalysis. Rather than representing entirely new sensing modalities, these approaches combine established principles to overcome limitations associated with individual technologies.

Several directions currently dominate the field. Plasmonic metasurfaces exploit subwavelength architectures engineered to tailor optical responses with unprecedented precision, while hybrid plasmonic-photonic systems couple plasmonic resonances with dielectric resonators or guided optical modes to enhance sensing performance. Another direction integrates nanoplasmonics with microfluidics and machine learning-assisted data analysis, enabling more compact and automated analytical workflows. More recently, polarisation-selective plasmonic architectures have introduced an additional degree of optical multiplexing, in which individually addressable nanoapertures or metasurface elements can be independently interrogated through their polarisation-dependent optical responses. This strategy increases information density without increasing device footprint and extends multiplexing beyond conventional spatial and spectral encoding. Although these concepts remain largely at the proof-of-concept stage and have primarily been demonstrated in nanophotonic systems, they represent a promising direction for future high-density multiplexed plasmonic biosensing platforms [31,32,33].

These approaches offer several attractive features, including enhanced sensitivity, improved optical quality factors, greater integration flexibility, and compatibility with miniaturised sensing platforms. Recent demonstrations span applications ranging from nucleic acid detection and EVs profiling to tumour-associated biomarker analysis, while continuing to expand into food and environmental analyses as well as portable sensing applications [34,35,36,37].

Despite these advances, most emerging nanoplasmonic platforms remain at relatively low levels of technological maturity. Challenges related to scalable nanofabrication, large-area reproducibility, standardisation, and management of increasingly high-dimensional datasets remain unresolved. Consequently, their near-term impact will likely be greatest in specialised applications rather than routine analytical practice.

The broader implementation of these emerging platforms will depend less on incremental improvements in analytical performance than on advances in scalable manufacturing, standardisation, regulatory acceptance, and seamless integration into routine analytical practice. The growing integration of deep learning and nanoplasmonic sensing is expected to contribute not only to data interpretation, but increasingly also to assay optimisation and multimodal data integration, particularly in EV research [38,39]. Emerging nanoplasmonic systems are therefore expected to integrate rather than replace established multiplexing strategies.

A unifying principle emerges from this discussion: despite their technological diversity, all multiplexed plasmonic platforms ultimately depend on the quality of the biointerface through which molecular recognition is achieved.

## 4. Biointerface Engineering for Multiplexed Plasmonic Biosensing

While optical instrumentation often receives most attention in plasmonic biosensing, successful multiplexing is increasingly determined by biointerface engineering, namely the ability to create robust, selective, and reproducible interfaces between biological recognition elements and plasmonic transducers. As multiplexing density increases, the sensor surface itself becomes a critical determinant of analytical performance. Multiple recognition elements must coexist on a single substrate without compromising selectivity, signal integrity, or assay reproducibility. Consequently, surface engineering should no longer be considered a secondary experimental step, but rather a central design parameter of multiplexed biosensing systems.

Four interdependent aspects underpin successful multiplexed biointerface engineering: surface biofunctionalisation, implementation of antifouling interfaces, diversification of molecular recognition strategies, and integration of microfluidic and microarray architectures. Together, these elements largely determine whether multiplexed plasmonic assays can progress from proof-of-concept demonstrations towards robust analytical platforms suitable for translational applications.

### 4.1. Surface Biofunctionalisation

Surface biofunctionalisation constitutes one of the fundamental prerequisites for multiplexed biosensing because it determines how multiple recognition elements are immobilised, organised, and maintained on a common sensing surface. Regardless of the optical modality employed, successful implementation requires precise localisation of capture probes while preserving their biological activity and minimising cross-reactivity between neighbouring sensing regions.

Several complementary approaches have been developed to achieve this objective. Robotic spotting remains one of the most widely adopted approaches and encompasses both contact and non-contact deposition methods, particularly piezoelectric dispensing and inkjet-based printing technologies. These approaches have been extensively applied to DNA, protein, lectin, and glycan microarrays because they enable reproducible fabrication of high-density sensing architectures while minimising sample consumption and facilitating automation [40]. Microcontact printing (μCP) offers a complementary strategy based on elastomeric stamps and is particularly attractive for generating well-defined biomolecular patterns with high spatial precision [41]. Microfluidics-assisted immobilisation provides an additional level of control by compartmentalising immobilisation steps and reducing the risk of cross-contamination between sensing regions. Such approaches are particularly attractive for multiplexed SPR and SPRi platforms because they facilitate integration of surface patterning with automated fluid handling and high-throughput assay workflows [42].

As multiplexing density increases, maintaining receptor activity becomes equally important as controlling receptor position. Random immobilisation often leads to unfavourable orientations that partially obstruct binding sites and reduce assay performance. Consequently, increasing attention has been devoted to oriented immobilisation strategies. DNA-directed immobilisation using Protein G–DNA conjugates represents one elegant example, enabling controlled antibody orientation while simultaneously increasing accessibility of binding sites through the introduction of molecular spacers [42,43].

More broadly, these examples illustrate a fundamental principle of multiplexed biosensing: increasing sensor density alone does not necessarily improve analytical performance. On the contrary, excessive probe crowding may reduce accessibility, introduce steric effects, and compromise reproducibility. Accordingly, optimisation of ligand density, immobilisation chemistry, and receptor orientation should be regarded as integral components of multiplexing design rather than as independent surface engineering tasks. Importantly, fabrication technologies should not be evaluated solely by achievable spot density, but also by their ability to maintain probe activity, inter-spot reproducibility, and compatibility with automated manufacturing processes.

### 4.2. Antifouling Interfaces

Non-specific adsorption remains one of the major obstacles to implementing multiplexed plasmonic biosensing in real-world samples. This challenge becomes particularly pronounced when analysing complex matrices such as serum, plasma, saliva, urine, milk, or minimally processed biological fluids, where abundant proteins can readily occupy available surface sites and generate false signals.

The consequences are amplified in multiplexed assays. Fouling not only increases background noise but may also introduce apparent signals in neighbouring sensing regions, thereby compromising quantitative analysis and reducing inter-channel independence. Effective antifouling strategies are therefore essential for preserving both signal integrity and multiplexing fidelity.

Zwitterionic materials have emerged as particularly attractive antifouling coatings because their balanced charge distribution promotes the formation of highly hydrated interfacial layers that effectively resist protein adsorption. Recent studies have demonstrated excellent antifouling performance of zwitterionic peptide hydrogels while simultaneously maintaining compatibility with clinically relevant biomarker detection in serum samples [44].

Mixed-charge architectures provide another promising solution. Poly-L-lysine-based interfaces combining antifouling oligopeptides with sparsely distributed recognition probes have enabled direct analysis of complex samples with minimal pre-analytical processing, illustrating the versatility of this approach beyond traditional biomedical applications [45].

A further important antifouling strategy is provided by hydrogels. Their highly hydrated three-dimensional structures can simultaneously suppress non-specific adsorption and provide high loading capacities for recognition elements. Recent developments have further expanded their applicability towards mechanically robust and tunable materials suitable for long-term biosensing applications [46,47].

These advantages, however, should be weighed against potential trade-offs. Increasing the thickness or hydration of antifouling layers may increase the distance between the sensing surface and target molecules, reduce mass transport efficiency, and limit accessibility of surface-immobilised recognition elements, particularly for large biological targets. Consequently, antifouling interfaces should be optimised not only to suppress non-specific adsorption but also to preserve efficient target transport, favourable binding kinetics, and analytical sensitivity [48,49].

Although poly (ethylene glycol) (PEG) remains widely used, its susceptibility to oxidative degradation has stimulated growing interest in alternative antifouling strategies. Increasing evidence suggests that zwitterionic materials and advanced hydrogel systems may offer superior long-term performance, particularly when multiplexed analyses are performed in complex biological matrices.

Ultimately, antifouling interfaces should no longer be considered optional surface modifications. They have become indispensable components of multiplexed bioanalysis and will likely determine the practical usability of future plasmonic platforms in translational applications.

### 4.3. Molecular Recognition Strategies

Antibodies remain the dominant recognition elements in plasmonic biosensing because of their high specificity and broad availability. However, several intrinsic limitations become increasingly apparent as multiplexing complexity grows. Batch-to-batch variability, limited long-term stability, relatively large molecular dimensions, and production costs have stimulated considerable interest in alternative recognition strategies.

Aptamers represent one of the most mature alternatives. These short nucleic acid sequences combine high affinity with small molecular size and excellent compatibility with dense array architectures. Their synthetic production further improves reproducibility and facilitates multiplexed assay development. Recent SPRi platforms have demonstrated simultaneous kinetic analysis of multiple aptamer–target interactions with high spatial densities and minimal cross-contamination, highlighting their potential for highly integrated biosensing systems [50].

SERS-based aptasensors have further illustrated the versatility of aptamers by enabling simultaneous detection of multiple biomarkers with exceptionally high sensitivity. Such systems combine the molecular specificity of aptamers with the spectral multiplexing capabilities of SERS, creating particularly attractive opportunities for future high-content analyses [51].

Molecularly imprinted polymers (MIPs) constitute another promising class of recognition elements. Unlike biological receptors, MIPs are entirely synthetic and can exhibit excellent chemical stability and long storage lifetimes. Recent epitope-imprinted nanoMIPs have demonstrated high affinity towards clinically relevant biomarkers while maintaining excellent selectivity against structurally unrelated proteins [52].

Lectins deserve particular attention because they provide access to a molecular dimension that remains difficult to interrogate using conventional affinity reagents. By recognising specific glycan motifs, lectins enable glycoprofiling of proteins, EVs, and other biological particles. Such capabilities are particularly relevant given the growing recognition that glycosylation signatures may contain clinically valuable information that extends beyond conventional proteomic measurements. Lectin-based analytical strategies have become an important tool for glycan biomarker discovery, while SPRi-based lectin arrays have demonstrated considerable potential for rapid, high-throughput glycan profiling and fingerprinting [53,54].

Importantly, no single recognition element is universally optimal. Instead, future multiplexed plasmonic platforms will likely rely on combinations of complementary recognition strategies selected according to target type, sample complexity, and analytical requirements. This transition from antibody-centric designs towards diversified recognition architectures may ultimately prove as important as advances in optical instrumentation itself.

### 4.4. Microfluidic and Microarray Integration

The evolution of multiplexed plasmonic biosensing increasingly depends on successful integration of complementary technologies instead of further improvements in optical sensitivity alone. Among these, microfluidics and microarray architectures have emerged as key enabling components because they address practical limitations associated with sample consumption, assay automation, throughput, and analytical reproducibility.

Microarrays provide a natural implementation of spatial multiplexing by enabling simultaneous interrogation of multiple recognition elements on a common sensing surface. Their compatibility with SPRi, LSPR, and SERS platforms has facilitated the transition from single-analyte measurements towards multidimensional molecular profiling. However, increasing array density does not automatically improve analytical performance, as higher densities also amplify the impact of cross-reactivity, non-specific interactions, and data-processing complexity.

Microfluidics addresses complementary challenges by improving reagent delivery, reducing sample consumption, and enabling automated workflows and temporal multiplexing strategies. These capabilities are particularly valuable for multiplexed SPR and SPRi platforms, where assay robustness and kinetic reproducibility are essential performance parameters [55].

Importantly, the greatest value of these technologies emerges when they are integrated into unified analytical workflows. Increasingly, future platforms are combining patterned sensor arrays, automated fluid handling, and computational analysis within compact lab-on-a-chip architectures. Instead of maximising multiplexing density alone, such systems seek to optimise the balance between information content, assay robustness, and operational simplicity [56]. Recent examples further demonstrate that such integration can be implemented in compact, multichannel plasmonic platforms capable of simultaneous analysis of multiple clinically relevant targets from a single sample, highlighting the growing translational maturity of multiplexed plasmonic biosensing [57].

Nevertheless, substantial challenges remain. Device fabrication often requires specialised manufacturing procedures, fluidic architectures become increasingly complex as multiplexing levels increase, and many integrated systems remain confined to proof-of-concept demonstrations. Consequently, future progress will likely depend less on introducing additional sensing channels and more on improving manufacturing reproducibility, interoperability between platform components, and compatibility with existing laboratory workflows. From a broader perspective, integration may ultimately represent the next major step in the evolution of multiplexed plasmonic biosensing. Future implementations of microfluidic–microarray integration are unlikely to result from advances in fluid handling or array fabrication alone, but from their coordinated optimisation within practical multiplexed analytical platforms.

These advances in biointerface engineering are ultimately valuable only insofar as they enable robust analytical performance in demanding real-world applications. The following section therefore considers representative application areas that currently drive the development of multiplexed plasmonic biosensing.

## 5. Application Areas Driving Multiplexed Plasmonic Biosensing

The growing interest in multiplexed plasmonic biosensing is largely driven by application areas that inherently require simultaneous interrogation of multiple molecular targets. In many biological systems, relevant information is distributed across combinations of proteins, nucleic acids, glycans, EVs, and biomolecular interaction patterns instead of being contained within a single biomarker. Consequently, these applications increasingly illustrate why multiplexing has become an analytical necessity in many areas of contemporary bioanalysis more than a technological luxury. The emphasis in recent plasmonic biosensing research has therefore shifted from maximising analytical sensitivity towards generating biologically relevant information from complex biological systems [15]. Although these application areas differ substantially in biological complexity and clinical maturity, they share a common analytical challenge: extracting multidimensional information from limited sample volumes while maintaining sufficient sensitivity, specificity, and assay robustness. Instead of providing an exhaustive overview of individual applications, this section highlights representative examples that illustrate where multiplexed plasmonic biosensing may offer unique analytical advantages and where important translational challenges remain.

### 5.1. Liquid Biopsy

Liquid biopsy has emerged as one of the strongest drivers for multiplexed biosensing because complex biological conditions rarely allow reliable characterisation using a single molecular marker. Instead, relevant information is increasingly derived from combinations of circulating proteins, nucleic acids, EVs, and complementary molecular signatures. Although oncology remains its most established application area, liquid biopsy is progressively expanding towards other diseases and monitoring applications, further increasing the demand for multidimensional analytical approaches [38].

Plasmonic biosensing is well suited to this context because it combines label-free analysis with low sample consumption and compatibility with multiplexed assay architectures. SPRi-based microarrays and nanoplasmonic platforms have demonstrated the ability to simultaneously interrogate multiple tumour-associated markers while preserving the possibility of real-time interaction analysis. Recent developments have also incorporated plasmonic metasurfaces and integrated nanoplasmonic architectures to analyse complex biomarker panels from small sample volumes. Representative examples include multiplexed plasmonic assays that simultaneously interrogate several circulating biomarkers and demonstrate how integrated molecular signatures can outperform isolated measurements [58,59].

Nevertheless, important limitations remain. Tumour heterogeneity varies substantially between patients, biomarker panels often lack standardisation, and many proposed platforms have only been evaluated in small cohorts. Therefore, future progress will depend primarily on establishing reproducible biomarker combinations and clinically validated workflows.

### 5.2. Glycoprofiling

Among representative applications of multiplexed biosensing, glycoprofiling most clearly illustrates why multiplexing is biologically necessary rather than merely analytically advantageous. Glycosylation itself is intrinsically multidimensional, and changes in glycan branching, sialylation, fucosylation, and site-specific glycosylation patterns frequently occur simultaneously and are difficult to capture using single-analyte approaches. Lectin arrays, glycan arrays, and orthogonal recognition strategies make plasmonic biosensing especially well suited to this application because it enables interrogation of multiple glycan motifs simultaneously. Such approaches can provide molecular fingerprints that extend beyond conventional measurements of protein abundance and reveal biologically relevant information that may otherwise remain inaccessible [60].

Multiplexed glycoprofiling is increasingly being explored in oncology, inflammatory diseases, and neurodegenerative disorders, where glycosylation signatures may complement established biomarkers. Importantly, these applications align particularly well with the strengths of plasmonic biosensing because glycan recognition often benefits from direct molecular detection and real-time monitoring of weak multivalent interactions [61].

Despite substantial progress, glycoprofiling remains challenging. Glycan heterogeneity is exceptionally high, lectin specificity is often overlapping, and universal standards remain largely unavailable. Consequently, future developments will require improved reference materials, better orthogonal validation strategies, and standardised analytical pipelines.

### 5.3. EVs Profiling

EVs exemplify one of the greatest analytical challenges in multiplexed biosensing: biological heterogeneity within individual particles and across vesicle populations. EVs simultaneously carry proteins, nucleic acids, lipids, and glycoconjugates, and single-marker approaches provide only a limited description of their biological origin and functional state. Multiplexed plasmonic biosensing offers several advantages in this area. Spatially resolved microarrays and nanoplasmonic platforms can simultaneously interrogate multiple EV surface markers while preserving compatibility with small sample volumes and minimally processed biological fluids [62].

Especially promising developments involve integrated profiling strategies that combine protein markers with glycosylation signatures or other orthogonal molecular descriptors. Such multidimensional approaches may improve EV classification and provide richer biological information than conventional particle-counting methods alone. However, EV analysis remains technically demanding. Pre-analytical variability, lack of universally accepted reference materials, and inconsistencies in isolation procedures continue to hamper inter-study comparability. As a result, analytical standardisation remains one of the major barriers to broader implementation.

### 5.4. Infectious Diseases and Syndromic Testing

Among the diverse application areas of multiplexed plasmonic biosensing, infectious disease diagnostics illustrate a fundamentally different analytical priority: rapid differential diagnosis. In many clinical scenarios, the principal challenge is not the detection of a single pathogen with maximum sensitivity, but the timely discrimination between multiple pathogens or host-response patterns that present with similar clinical manifestations. Multiplexed plasmonic biosensing is therefore well suited because it enables simultaneous interrogation of multiple targets within a single analytical workflow, thereby supporting faster and more informed clinical decision making.

Multiplexed plasmonic biosensing is well positioned to contribute to this field because it combines rapid analysis with compatibility for high-density assay architectures. Among plasmonic approaches, SERS-based platforms are especially well suited because their narrow spectral fingerprints allow simultaneous detection of multiple pathogens or host-response markers within a single assay [63]. Recent studies have further explored integrated platforms capable of combining pathogen detection with inflammatory biomarkers, illustrating a gradual transition from single-pathogen detection towards multidimensional host–pathogen profiling [64,65]. Nevertheless, important translational challenges remain. Pathogen diversity, emergence of new variants, and regulatory requirements impose additional constraints on assay design and validation. Moreover, the diagnostic value of multiplexing must ultimately be balanced against operational simplicity if rapid syndromic testing is to become feasible outside specialised laboratories.

### 5.5. Emerging Non-Clinical Applications

The biomedical applications discussed above illustrate an important shift within biosensing. The primary objective is no longer to maximise the number of detectable analytes or analytical sensitivity, but to extract biologically relevant information from multidimensional molecular signatures. This objective is also well illustrated by non-clinical applications, which show that the conceptual advantages of multiplexing extend beyond medicine and arise whenever complex samples require simultaneous interpretation across multiple analytical dimensions. In this context, multiplexed plasmonic biosensing may offer unique opportunities by combining label-free analysis, compatibility with complex biological samples, and integration with high-content analytical workflows.

Although biomedical diagnostics remain the principal application area of multiplexed plasmonic biosensing, the analytical concepts underlying multiplexing are equally relevant to a wide range of non-clinical applications [37]. In these settings, the primary objective is often not simply to increase analytical throughput, but to obtain integrated information from multiple chemically or biologically distinct targets within complex sample matrices, thereby supporting more reliable assessment of sample quality, safety, and environmental status.

Food safety represents one of the most promising areas for broader implementation of multiplexed plasmonic biosensing. Contemporary food analysis increasingly requires the simultaneous determination of microbiological, chemical, and allergenic hazards that may coexist within a single sample. Representative examples include foodborne pathogens such as *Salmonella*, *Listeria monocytogenes*, and *Escherichia coli* O157:H7, mycotoxins including aflatoxin B1 and ochratoxin A, food allergens, and residues of veterinary drugs. Simultaneous detection of multiple foodborne pathogens has already been demonstrated using multichannel SPR platforms, while more recent metasurface plasmon resonance and SERS-based assays have expanded multiplexed analysis to antibiotic residues and multiple mycotoxins in complex food matrices. Collectively, these studies demonstrate how multiplexed plasmonic biosensors can integrate the detection of microbiological and chemical hazards within a single analytical workflow, reducing sample consumption and analytical time while providing more comprehensive information than sequential single-analyte testing [4,66,67,68,69,70,71].

Comparable analytical demands arise in environmental monitoring, where water and environmental samples frequently contain complex mixtures of chemically diverse contaminants requiring simultaneous assessment. Multiplexed plasmonic sensing has therefore attracted increasing interest for the parallel determination of pesticide residues, cyanobacterial toxins such as microcystin-LR, endocrine-disrupting compounds, and per- and polyfluoroalkyl substances (PFAS), particularly in combination with portable sensing platforms, integrated microfluidics, and miniaturised analytical devices suitable for on-site monitoring. Although most reported systems remain at the proof-of-concept stage and require further validation before routine deployment, they demonstrate that the principal barriers to translation are increasingly associated with assay robustness, standardisation, and analytical validation rather than with multiplexing capability itself [4].

These examples demonstrate that the analytical principles underpinning multiplexed plasmonic biosensing are broadly transferable across biomedical and non-clinical applications, although their practical implementation must be adapted to the specific requirements of each field. The same design principles discussed throughout this Feature Paper, including robust surface biofunctionalisation, appropriate assay design, standardised analytical workflows, and reliable data interpretation, are expected to underpin successful implementation of multiplexed plasmonic biosensing across biomedical, food, environmental, and other analytical applications.

Although these examples illustrate the practical value of multiplexed plasmonic biosensing, they also highlight that plasmonic approaches represent only one part of a broader optical biosensing landscape.

## 6. Positioning Plasmonic Biosensing Within the Broader Optical Multiplexing Landscape

Multiplexed plasmonic biosensing has evolved alongside several other multiplexed optical technologies capable of simultaneous multi-analyte detection. Consequently, its analytical value can only be fully appreciated when considered within the broader landscape of multiplexed bioanalytical platforms. Instead of competing directly with established fluorescence-based and interferometric platforms, plasmonic biosensing occupies a complementary analytical position defined by its combination of label-free detection, real-time interaction monitoring, surface-confined sensing, and compatibility with multiplexed assay architectures. This section therefore places plasmonic biosensing into the wider context of multiplexed optical technologies and critically discusses where its unique analytical capabilities provide genuine added value.

### 6.1. Non-Plasmonic Optical Multiplexing Technologies

Fluorescence-based microarrays and bead-based immunoassays remain among the most widely adopted multiplexed analytical platforms in biomedical research and clinical laboratories. Their principal strengths include excellent analytical sensitivity, mature instrumentation, well-established workflows, and the ability to quantify large biomarker panels with high throughput. These characteristics have established fluorescence-based technologies as reference platforms for multiplexed protein analysis and numerous research and diagnostic applications [72].

Chemiluminescence and electrochemiluminescence (ECL) assays provide similar analytical advantages, combining broad dynamic range with excellent sensitivity and robust quantitative performance. These approaches rely on labelled detection reagents and typically provide endpoint measurements without direct access to biomolecular interaction kinetics or affinity information [73].

FRET-based biosensors further extend fluorescence-based analysis by enabling highly sensitive, distance-dependent monitoring of molecular proximity, conformational changes, and biomolecular interactions. Multiplexed FRET configurations can be implemented using spectrally distinguishable donor–acceptor pairs or multi-FRET architectures, although their multiplexing capacity is constrained by spectral overlap, cross-talk, and the complexity of probe design. Unlike label-free plasmonic methods, FRET requires fluorescent donor and acceptor components, but it can provide real-time and time-resolved information on molecular events [74].

Among label-free optical methods, interferometric techniques, particularly biolayer interferometry (BLI), represent one of the closest analytical counterparts to plasmonic biosensing. BLI is well established for biomolecular interaction analysis, antibody characterisation, and biopharmaceutical development owing to its relatively simple instrumentation and robust assay implementation. Conventional BLI instruments generally provide lower multiplexing density than imaging-based SPR or planar microarray formats and can be less sensitive to low-molecular-weight analytes, depending on assay configuration and instrument design. Nevertheless, they retain the principal advantages of label-free, real-time kinetic analysis [73].

### 6.2. Comparative Characteristics of Optical Multiplexing Platforms

No single optical technology simultaneously maximises analytical sensitivity, multiplexing capacity, real-time interaction analysis, ease of implementation, and translational maturity. Instead, each platform addresses a distinct combination of analytical requirements and therefore occupies a different position within the multiplexed biosensing landscape. The key characteristics of representative optical multiplexing platforms are summarised in Table 3. The qualitative ratings are intended to provide a comparative conceptual overview of representative platform characteristics rather than absolute performance rankings. Actual analytical performance varies with platform configuration, assay design, target class, and experimental implementation. The comparison emphasises that selecting an appropriate optical multiplexing platform should be guided by the analytical question and the biological information required, instead of by multiplexing capacity alone.

Label-based technologies currently dominate applications requiring large multiplexing panels, ultra-high analytical sensitivity, and highly standardised workflows. Their widespread commercial adoption reflects decades of technological optimisation, robust validation, and compatibility with routine laboratory operation. However, the requirement for labelled detection inevitably increases assay complexity and precludes direct observation of biomolecular interaction kinetics [75].

Interferometric and plasmonic biosensing share several important analytical characteristics, particularly with respect to label-free detection and real-time kinetic analysis. However, the broader plasmonic family considered in this Feature Paper extends beyond conventional SPR to include SPRi, LSPR, and SERS, thereby encompassing multiplexing strategies that integrate spatial, spectral, and surface-sensitive analytical capabilities thereby extending the analytical scope from interaction analysis towards integrated multiplexed molecular profiling.

Plasmonic biosensing follows a different analytical philosophy. Rather than maximising the number of detectable analytes alone, SPR, SPRi, LSPR, and SERS seek to combine multiplexed detection with additional layers of analytical information, including interaction kinetics, affinity, molecular recognition specificity, and surface-confined biomolecular processes. These complementary data can substantially enrich biological interpretation in applications where understanding molecular interactions is as important as quantification itself.

Suspension-array technologies, such as bead-based multiplex immunoassays, occupy an intermediate position by providing robust quantitative analysis of moderately to highly multiplexed biomarker panels with excellent throughput and reproducibility, but without direct access to interaction kinetics or surface binding phenomena [76].

Taken together, these observations suggest that multiplexing should no longer be evaluated solely by the number of simultaneously detectable analytes. The key question is not how many analytes can be measured, but what biologically meaningful information can be extracted in a robust, reproducible, and ultimately translatable manner. From this perspective, multiplexing capacity represents only one dimension of analytical performance rather than an objective in itself. Table 3 illustrates that different multiplexing technologies provide distinct combinations of analytical capabilities and should therefore be regarded more as complementary than competing analytical platforms.

### 6.3. Current Position of Plasmonic Biosensing

Current multiplexed molecular profiling is largely supported by several mature analytical platforms, including bead-based suspension arrays (e.g., Luminex), proximity extension assays (Olink), and aptamer-based proteomic platforms (SomaScan). These technologies have achieved a high degree of analytical standardisation and commercial maturity, particularly for high-throughput protein profiling [77,78]. Rather than directly competing with these established platforms, multiplexed plasmonic biosensing offers complementary capabilities whenever label-free detection, real-time interaction analysis, or integrated molecular profiling provide additional analytical value. Despite remarkable progress in plasmonic sensor development, multiplexed plasmonic biosensing has not yet reached the level of routine implementation achieved by fluorescence-based multiplex immunoassays, bead-based suspension arrays, or nucleic acid analysis platforms. Importantly, this difference is no longer explained primarily by insufficient analytical performance. Instead, it reflects broader challenges related to reproducibility, biointerface engineering, assay standardisation, data interpretation, manufacturing, and large-scale validation, issues that will be discussed in the following section.

Instead of replacing established multiplexed technologies, plasmonic biosensing is increasingly emerging as a distinctive analytical platform for applications that uniquely benefit from label-free detection, real-time interaction monitoring, and multidimensional molecular characterisation. Representative examples include biomolecular interaction studies, affinity determination, glycoprofiling, EV analysis, small-molecule detection, and advanced liquid biopsy applications, where complementary kinetic and molecular information can provide additional biological insight beyond endpoint quantification alone. Comparable advantages may also be realised in non-clinical settings, including food safety and environmental monitoring, where simultaneous label-free analysis of multiple targets can complement established analytical workflows. Beyond optical biosensing, multiplexed molecular analysis is increasingly complemented by nucleic acid technologies such as multiplex PCR and next-generation sequencing, particularly where genomic or transcriptomic information is required.

Recent developments further illustrate this evolution. Highly engineered plasmonic nanostructures have demonstrated exceptional analytical performance for low-molecular-weight analytes and ultra-low analyte concentrations, expanding the capabilities of label-free plasmonic sensing beyond those of conventional SPR architectures [79]. At the same time, increasing integration with microfluidics, nanophotonics, automated biointerface engineering, and artificial intelligence is gradually transforming plasmonic biosensors from individual sensing devices into integrated analytical systems capable of addressing complex multiplexed workflows [80].

Consequently, the future impact of multiplexed plasmonic biosensing is unlikely to be determined solely by further increases in multiplexing capacity or analytical sensitivity. Instead, its greatest strength is expected to lie in integrating multiple layers of molecular information within robust, reproducible, and application-oriented analytical workflows. Regardless of the sensing modality employed, successful implementation ultimately depends on overcoming a common set of analytical and translational challenges.

## 7. Challenges Towards Reproducibility, Standardisation, and Translation

Despite remarkable advances in plasmonic materials, sensor architectures, nanofabrication, and analytical performance, relatively few multiplexed plasmonic biosensors have progressed beyond proof-of-concept demonstrations. While early research primarily focused on improving sensitivity and increasing multiplexing capacity, the principal challenges have gradually shifted towards reproducibility, analytical validation, and practical implementation [5,6,38]. Today, further improvements in sensor performance alone are unlikely to accelerate translation unless accompanied by robust biointerface engineering, harmonised analytical protocols, and reproducible manufacturing strategies. Consequently, successful implementation should be viewed as a systems-level challenge encompassing the entire analytical workflow rather than the sensing element alone.

### 7.1. Robust Biointerfaces and Reproducible Sensor Performance

The analytical performance of multiplexed plasmonic biosensors ultimately depends on the quality and reproducibility of the biointerface, not only on optical transduction alone. Variability introduced during nanostructure fabrication, surface functionalisation, immobilisation of recognition elements, and nonspecific adsorption often exceeds the intrinsic variability of the optical detection system itself [81]. These effects become increasingly pronounced as multiplexing density increases, where even minor differences between sensing regions may compromise quantitative comparison across the array.

Surface biofunctionalisation strategies discussed in Section 4 therefore represent not only enabling technologies but also one of the principal determinants of analytical robustness. Stable probe orientation, controlled immobilisation density, efficient antifouling coatings, and long-term surface stability all directly influence assay reproducibility. Similar challenges apply across SPR, SPRi, LSPR and SERS platforms, although their relative importance varies according to the sensing modality. For example, SERS additionally requires reproducible generation of plasmonic hotspots, whereas SPR-based platforms are particularly sensitive to variability in ligand immobilisation and mass transport conditions [82].

Another practical consideration, particularly for nanoplasmonic platforms operating under high optical excitation, is local plasmonic heating. Local temperature gradients may influence binding kinetics, local refractive index, and biomolecular behaviour, thereby affecting analytical performance. Although such effects are generally negligible under conventional SPR and SPRi operating conditions, they should be considered during the design of nanoplasmonic biosensors employing strong local field enhancement [83].

These observations suggest that future improvements should focus less on maximising analytical sensitivity and more on engineering robust, reproducible biointerfaces capable of maintaining consistent performance across multiple fabrication batches and independent laboratories.

### 7.2. Standardisation and Analytical Validation

Analytical reproducibility extends beyond sensor fabrication. The absence of harmonised validation procedures, commutable reference materials, standard reporting practices, and inter-laboratory benchmarking remains one of the major barriers preventing objective comparison between multiplexed plasmonic biosensors developed by different research groups [84,85,86]. The situation is further complicated by the absence of universally accepted performance metrics and minimum reporting requirements, limiting independent validation and meaningful comparison across studies.

Unlike established analytical technologies, where validation protocols and performance metrics are well defined, multiplexed plasmonic assays are frequently evaluated using different sample types, analytical endpoints, immobilisation strategies, and data processing methods. Consequently, reported improvements in sensitivity or multiplexing capacity often cannot be directly compared across studies.

Progress towards practical implementation will therefore depend as much on analytical validation as on continued technological innovation. The development of commutable reference materials, standardised assay protocols, harmonised reporting guidelines, and multicentre performance studies will be essential for establishing confidence in multiplexed plasmonic measurements and enabling meaningful comparison across independently developed platforms.

Although these requirements are particularly well recognised in biomedical and clinical research, comparable validation principles are equally important for food safety, environmental monitoring, and other non-clinical applications, where analytical comparability and regulatory confidence are likewise essential.

### 7.3. Clinical Translation and Regulatory Implementation

Clinical implementation introduces requirements that extend well beyond analytical performance. In addition to demonstrating sensitivity and specificity, multiplexed biosensors must provide robust manufacturing processes, batch-to-batch reproducibility, quality control procedures, long-term stability, clinically validated workflows, and compliance with regulatory frameworks. These requirements are common to all in vitro diagnostic technologies but become increasingly demanding as assay complexity increases [86,87].

Importantly, the limited clinical adoption of multiplexed plasmonic biosensors should not be interpreted as evidence of insufficient analytical capability. Instead, it largely reflects the fact that many published studies remain focused on demonstrating novel sensing concepts rather than establishing comprehensive validation pathways comparable to those successfully developed for mature multiplexed technologies. Existing commercial multiplex technologies demonstrate that successful implementation depends on much more than analytical performance alone. Reproducibility, standardisation, manufacturing scalability, software integration, and regulatory acceptance are equally important prerequisites for routine deployment [6,38].

**Figure 3:**
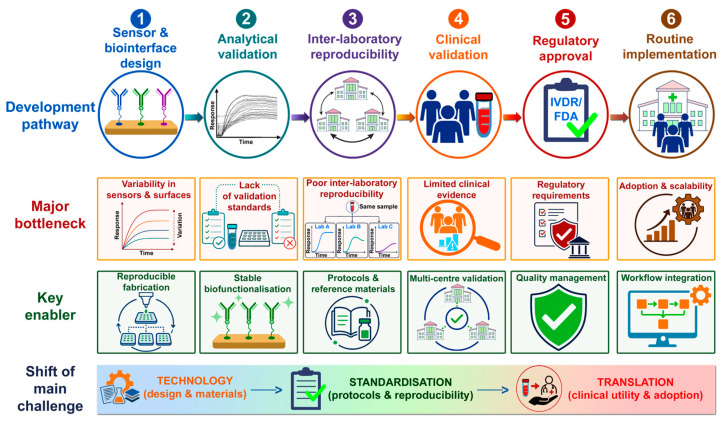
Translational bottlenecks in multiplexed plasmonic biosensing.

Consequently, future translational efforts should place greater emphasis on multicentre validation, quality assurance, standard operating procedures, and integrated analytical workflows that can be implemented reproducibly outside highly specialised research laboratories. Accordingly, the principal barriers to translation are no longer technological but organisational, analytical, and regulatory. Figure 3 illustrates the sequential progression from sensor design and biointerface engineering through analytical validation, inter-laboratory reproducibility, clinical validation, and regulatory approval towards routine implementation, highlighting the principal bottleneck and representative enabling strategies at each stage.

### 7.4. Towards Practical Implementation

The analysis presented throughout this Feature Paper indicates that successful implementation of multiplexed plasmonic biosensing will depend less on achieving ever higher multiplexing levels or lower limits of detection than on the reliable translation of technological advances into reproducible analytical systems encompassing surface engineering, assay standardisation, data analysis, quality assurance, and validation.

Successful translation will require the early integration of materials science, surface chemistry, analytical chemistry, microfluidics, data science, manufacturing, and regulatory expertise, making validation and standardisation integral design principles rather than downstream activities. The value of multiplexing should not be judged solely by the number of simultaneously detectable analytes, but by the robustness, reproducibility, and biological relevance of the information delivered to the end user.

These challenges should not be viewed merely as limitations but as the principal directions along which the next generation of multiplexed plasmonic biosensors will evolve.

## 8. Future Outlook and Opportunities

### 8.1. From Better Sensors to Better Systems

Over the past two decades, plasmonic biosensing has evolved from individual sensing technologies into increasingly integrated multiplexed analytical platforms. Building on the challenges discussed in Section 7, the future development of multiplexed plasmonic biosensing will increasingly depend on the integration of sensing technologies into complete analytical systems capable of delivering reproducible and application- relevant information [5,38]. Future multiplexed biosensors will therefore need to combine robust surface biofunctionalisation, automated sample handling, microfluidics, advanced data processing, and standardised analytical workflows within a single platform. Such integration will facilitate the transition of multiplexed plasmonic biosensors from research tools towards robust analytical systems suitable for biomedical research, pharmaceutical development, and eventually routine clinical implementation [6].

### 8.2. Data-Driven and AI-Assisted Biosensing

Multiplexed assays generate increasingly complex, high-dimensional, and potentially nonlinear analytical datasets. Artificial intelligence (AI), particularly machine learning (ML), is increasingly being applied to multiplexed biosensing workflows as a complementary analytical tool rather than an alternative to sensing technologies. Their principal contribution lies in the extraction of biologically meaningful information from multidimensional datasets generated by multiplexed assays, particularly where conventional statistical approaches, linear unmixing, or manual interpretation become insufficient [88,89].

Potential applications include automated signal processing, spectral deconvolution, feature extraction, image analysis, biomarker selection, pattern recognition, and multimodal data integration. In SERS and related vibrational plasmonic techniques, ML can facilitate analysis of partially overlapping spectral signatures and classification of complex spectral datasets. In SPRi, AI-assisted analysis can support automated evaluation of large imaging and kinetic datasets, including spot segmentation, drift correction, quality control, and classification of binding events. ML may also facilitate optimisation of assay design, surface functionalisation strategies, and quality control by identifying systematic sources of analytical variability that are difficult to recognise using conventional analytical methods [39,89].

However, AI cannot compensate for poor experimental design, inadequate surface chemistry, irreproducible sensor fabrication, or insufficient analytical validation. Reliable ML models ultimately depend on sufficiently large, diverse, and well-annotated datasets generated using reproducible analytical workflows. Overfitting, limited external validation, and insufficient model interpretability may further restrict broader implementation, particularly in clinical applications [88,90]. Consequently, advances in data science should be regarded as complementary to, rather than substitutes for, robust biointerface engineering, analytical standardisation, and rigorous experimental validation.

### 8.3. Design Principles for Future Multiplexed Biosensors

The preceding discussion suggests several general principles that may guide the future development of multiplexed plasmonic biosensors.

First, multiplexing strategies should be driven by biological questions instead of by maximising the number of simultaneously detectable analytes. The analytical value of multiplexing depends primarily on the biological relevance of the information obtained rather than on panel size alone.

Second, robust surface biofunctionalisation should be regarded as a prerequisite for reliable multiplexing more than an independent technological component. Improvements in reproducibility frequently contribute more to practical performance than further increases in analytical sensitivity.

Third, multiplexed biosensors should be designed as integrated analytical workflows that consider sensing, sample preparation, microfluidics, data processing, AI-assisted analysis and interpretation, and quality control together, not as independent modules.

Finally, standardisation and validation should be incorporated from the earliest stages of biosensor development. Designing with future reproducibility, scalability, and regulatory implementation in mind will substantially facilitate subsequent translation into practical applications.

### 8.4. Priorities for Standardisation

Although universally accepted standards for multiplexed plasmonic biosensing have yet to be established, several priorities are becoming increasingly apparent. These include the development of commutable reference materials, harmonised validation protocols, minimum reporting requirements, inter-laboratory benchmarking, and FAIR-compliant reference datasets suitable for evaluating analytical performance and validating machine learning algorithms [84,91].

Equally important will be the establishment of common performance metrics extending beyond analytical sensitivity and multiplexing capacity to include reproducibility, robustness, biological relevance, and clinical utility. Together with FAIR-compliant reference datasets, such frameworks would facilitate objective comparison across independently developed biosensors while supporting benchmarking and validation of machine learning algorithms [91].

### 8.5. Conclusions

Multiplexed plasmonic biosensing has evolved from a collection of individual sensing technologies into a diverse analytical platform encompassing SPR, SPRi, LSPR, SERS, and emerging hybrid approaches. Collectively, these modalities provide complementary capabilities that extend beyond conventional biomarker quantification by integrating label-free detection, real-time interaction analysis, molecular profiling, and multidimensional data generation within multiplexed assay formats.

At the same time, the major challenges facing the field have shifted from improvements in optical sensitivity towards reproducibility, standardisation, analytical validation, and practical implementation. Continued progress will therefore depend less on the development of increasingly sophisticated sensing elements than on the establishment of robust analytical systems integrating biointerface engineering, microfluidics, data science including AI-assisted data interpretation, quality assurance, and harmonised validation procedures.

Ultimately, the future impact of multiplexed plasmonic biosensing will be determined not by the number of simultaneously detectable analytes, but by its ability to generate reliable, reproducible, and biologically meaningful information that supports robust analytical and clinical decision-making. Achieving this goal will require robust, standardised analytical systems capable of translating technological advances into routine biomedical, environmental, and food analytical applications.

## Figures and Tables

**Table 1:** From Single-Analyte Measurements to Systems-Level Molecular Profiling: Key Analytical Drivers for Multiplexing.

Limitation	Consequence
Incomplete representation of biological complexity	Single biomarkers insufficiently capture multidimensional biological states
Limited analytical robustness	Increased susceptibility to false-positive and false-negative classifications
Missing systems-level information	Biomolecular interactions and network behaviour remain unresolved
Inefficient utilisation of biological samples	Multiple independent assays consume valuable specimens
Limited dynamic characterisation	Temporal evolution of biological processes is difficult to capture

**Table 2:** Multiplexing Paradigms Across Plasmonic Biosensing Platforms.

Platform	Dominant Multiplexing Strategy	Typical Multiplexing Scale	Major Strengths	Principal Limitations	Technology Maturity
SPR	Spatial, microfluidic	Few targets/channels	Robust kinetics, mature instrumentation	Limited multiplexing density	High
SPRi	Spatial	Tens–hundreds of sensing regions	True parallelisation	Optical complexity	High
LSPR	Spatial, spectral	Tens to hundreds of independently addressable sensing elements; potentially higher with hybrid encoding	Miniaturisation, portability	Nanofabrication variability	Moderate
SERS	Spectral	>20 spectrally resolved reporters demonstrated	Exceptional multiplexing capacity	Reproducibility	Moderate
Emerging nanoplasmonics	Hybrid	Application-dependent; potentially higher	Integration flexibility	Lack of standardisation	Low—moderate

Note: The reported practical multiplexing scales should be interpreted as representative orders of magnitude rather than directly comparable numerical metrics because different plasmonic modalities implement multiplexing through fundamentally different mechanisms (e.g., sensing channels, spatially resolved sensing regions, or spectrally distinguishable reporters).

**Table 3:** Comparative Characteristics of Representative Optical Multiplexing Platforms.

Characteristic	Fluorescence/Chemiluminescence (ECL)	Interferometry (BLI)	Plasmonics
Detection principle	Label-based	Label-free	Label-free
Real-time kinetic analysis	Low	High	High
Multiplexing capability	High	Low—Moderate	Moderate—High
Surface interaction analysis	Low	High	High
Analytical information content	Moderate	High	High
Standardisation maturity	High	High	Moderate
Primary application	High-throughput quantification	Interaction analysis	Interaction analysis, biomolecular profiling

Note: The qualitative categories are intended solely for comparative interpretation. Because different optical biosensing technologies optimise different analytical objectives (e.g., multiplexing capacity, kinetic information, sensitivity, analytical robustness, or translational maturity), no universally applicable numerical thresholds can be assigned to these categories.

## Data Availability

No new data were created or analysed in this study. Data sharing is not applicable to this article.
